# Systematically analyzed molecular characteristics of lung adenocarcinoma using metabolism-related genes classification

**DOI:** 10.1590/1678-4685-GMB-2022-0121

**Published:** 2023-01-06

**Authors:** Xiaoming Huang, Feng Zhang, Junqi Lin, Shaoming Lin, Guanle Shen, Xiaozhu Chen, Wenbiao Chen

**Affiliations:** 1 The Affiliated Hospital of Southern Medical University, People’s Hospital of Longhua, Department of respiratory medicine, Shenzhen, China.; 2 The First Affiliated Hospital of Jinan University, Department of Intensive Care Unit, Guangzhou, China.; 3 The Affiliated Hospital of Southern Medical University, People’s Hospital of Longhua, Department of Medical Ultrasound Department, Shenzhen, China.

**Keywords:** Lung adenocarcinoma, metabolic gene, molecular characteristics, metabolic classification, molecular classification

## Abstract

High heterogeneity of lung adenocarcinoma (LUAD) is a major clinical challenge. This study aims to characterize the molecular features of LUAD through classification based on metabolism-related genes. A total of 500 LUAD samples from The Cancer Genome Atlas (TCGA) and 612 from Gene Expression Omnibus (GEO) were integrated with 2,753 metabolism-related genes to determine the molecular classification. Systematic bioinformatics analysis was used to conduct correlation analysis between metabolism-related classification and molecular characteristics of LUAD. LUAD patients were divided into three molecular clusters (C1-C3). Survival analysis revealed that C1 and C2 showed good and poor prognoses, respectively. Associational analysis of classification and molecular characteristics revealed that C1 was associated with low pathological stage, metabolic pathways, high metabolic process, active immune process and checkpoint, sensitive drug response, as well as a low genetic mutation. Nevertheless, C2 was associated with high pathological stage, carcinogenic pathways, low metabolic process, inactive immune signatures, resistant drug response, and frequent genetic mutation. Eventually, a classifier with 60 metabolic genes was constructed, confirming the robustness of molecular classification on LUAD. Our findings promote the understanding of LUAD molecular characteristics, and the research data may be used for providing information be helpful for clinical diagnosis and treatment.

## Introduction

Lung cancer is a malignant tumor with the highest morbidity and mortality, and lung adenocarcinoma (LUAD) is its primary subtype (Torre et al., 2015). LUAD has a long incubation period, mild early symptoms, and a high degree of malignancy; besides, the affected patients are diagnosed at an advanced stage, hence missing the optimal time for treatment (Butnor, 2020). Research on the molecular mechanism of LUAD has advanced in the past decade; novel diagnostic methods, including targeting and immunotherapy, have caused substantial breakthroughs in clinical diagnosis and treatment (Collisson et al., 2014). Nonetheless, LUAD still faces hurdles, including postoperative recurrence, low drug sensitivity, and poor prognosis (Seguin et al., 2022); thus, there is an urgent need to improve the diagnostic level and treatment of LUAD. Notably, routine pathological staging determines treatment; however, there is a lack of multidisciplinary integration due to the radiological, pathological, and molecular heterogeneity of LUAD (Socinski et al., 2016). With the advent of sequencing technology, research and treatment of lung adenocarcinoma has expanded from purely histopathological subtype-based to molecular classification (Kashima et al., 2019). During the initiation and development of LUAD, different driver gene variants interact with each other to mediate tumor evolution; this is reflected in tumor heterogeneity (Inamura, 2018). Therefore, molecular classification of LUAD, integrated with clinicopathological characteristic analysis optimize the personalized treatment of LUAD patients.

Metabolic abnormality is a feature of tumor cells. Metabolic reprogramming in tumor cells maintains survival and infinite proliferation in a harsh microenvironment (Li and Zhang, 2016). Thus, abnormal metabolic pathways, regulatory molecules, and enzymes in tumor cells are vital targets for molecular classification (Khan et al., 2020). Based on the characteristics of glucose metabolism and immune cell infiltration in tumor microenvironment from transcriptome data of lung adenocarcinoma, Choi and Na (2018) divided LUAD patients into four clusters, showing different immune cell composition, tumor metabolism, and survival (Choi and Na, 2018). Goodwin et al. (2017) discovered distinct glycolytic metabolism-phenotype between lung squamous cell carcinoma (SqCC) and LUAD, with increased cellular glucose transporter molecules in SqCC at mRNA and protein levels but was lowest expressed in LUAD (Goodwin *et al.*, 2017). The above research suggests the possibility of performing LUAD classification from a metabolic perspective to identify distinct molecular characteristics and clinical features. Gene is a critical target for regulating metabolic reprogramming of tumor cells; it only participates in metabolic signaling pathways and regulates metabolic enzymes but is also regulated by non-coding RNA immune microenvironment and epigenetic modifications (Carrer and Wellen, 2015). Besides, the genes are prone to mutation and copy number variation (CNV) that constantly influences the metabolic process (Ried et al., 2019). In this work, we collected the metabolism-related genes from previous research and integrated them with LUAD samples for molecular classification. 

Datasets from the Gene Expression Omnibus (GEO) and The Cancer Genome Atlas (TCGA) databases were used to divide LUAD samples into a molecular classification based on metabolism-related genes; the LUAD samples were classified into three clusters (C1-C3) with significantly variant molecular characteristics and clinical features. Integrative analysis between three clusters and molecular characteristics revealed that C1 with good prognosis was associated with low pathological stage, metabolic pathways, high metabolic process, active immune process and checkpoint, sensitive drug response, and low genetic mutation. However, C2 with poor prognosis was related to high pathological stage, carcinogenic pathways, low metabolic process, low immune signatures, resistant drug response, and frequent genetic mutation. Moreover, a 60 metabolism-related genes classifier was established to confirm the classification efficacy. Our study suggests the possibility of classifying LUAD patients into three clusters based on metabolism-related gene subtypes. These three clusters may provide information be helpful for clinical diagnosis and treatment. 

## Methods

### Data selection and preprocessing

Raw data with clinical information was extracted from multiple databases, including The Cancer Genome Atlas (TCGA), Gene Expression Omnibus (GEO), on April 23, 2021. The processing process of GEO as following: CEL files with GSE19188, GSE29013, GSE30219, GSE31210, GSE37745, GSE50081 were downloaded from the GEO database, which retained samples of the above six datasets in GPL570 chip ([HG-U133_Plus_2] Affymetrix Human Genome U133 Plus 2.0 Array). Then, the RMA (Robust Multi-Array Average expression measure) function was used to process the profiling data by affy R package. Subsequently, the expression profile of the dataset was obtained through normalization. After eliminating the batch effects by the combat function of the sva R package, the six datasets were merged into one database, i.e., GSEdat. Principal components analysis (PCA) was used to access the elimination of batch effects across datasets. We converted the Annotation file probe of the merged datasets was converted to gene symbol based on GPL570. When multiple probes corresponded to the same gene symbol, the median value was considered the expression profile of the gene symbol, whereas the probe expression was eliminated when a probe corresponded to multiple gene symbols. The processing process of RNA-sequence from TCGA database as following: the FPKM expression spectrum data of primary tumor samples were downloaded from TCGA Data. The downloaded data were standardized read counts, and then converted into TPM with the code written by myself, followed by log2 conversion of the data. The single nucleotide variants from [MuTect2. Variant0. Maf] were obtained from TCGA. For clinical data, samples without survival time and status were excluded; as a result, 500 LUAD samples from TCGA and 612 from GEO were obtained for further analysis. The study was approved by the People’s Hospital of Longhua, Shenzhen. The statistical information of the filtered samples is shown in Table S1. The research flow chart is shown in Figure S1. 

### Metabolism-related genes classification of LUAD

In total, 2,752 previously reported metabolism- related genes involved in all metabolic processes were used in molecular classification analysis (Possemato et al., 2011). First, 2,752 metabolism-related genes with median absolute deviation (MAD) values (MAD≤0.5) were excluded from TCGA LUAD samples. Thereafter, univariate Cox analysis on metabolism-related genes was conducted using the coxph function of survival R package; *p* <0.05 was considered the threshold for filtering. Genes with significant prognostic value and MAD>0.5 were selected for classified analysis. Consequently, 408 metabolism-related genes were obtained for molecular typing (Table S2). Molecular typing was conducted using the ConsensusClusterplus R package to select the classification number; the k value determined the optimal number of clusters. Then, the mRNA expression data of these metabolism-related genes were used to confirm the subtype allocation using the T-distribution-based random neighbor embedding (T-SNE) algorithm. Meanwhile, LUAD samples from GSEdat were used to perform molecular classification analysis via similar methods. Gene Pattern category mapping (Submap) algorithm, a method evaluating the similarity between molecular classes based on expression profiles of independent sample cohorts, was conducted to ascertain whether the molecular typing of TCGA was similar to that of GSEdat. 

### Functional analysis of LUAD classification

The Limma R package was used to calculate differential expressed genes (DEGs) across all the subtypes, and false discovery rate (FDR) < 0.05 and an absolute value of fold change (FC) > 1.5 were set as the threshold to determine the significant DEGs. Subsequently, functional analysis of DEGs was conducted through Kyoto Encyclopedia of Genes and Genomes (KEGG) analysis using clusterprofiler R package with a significance threshold of FDR <0.05. 

### Metabolic characteristics of LUAD classification

A total of 113 metabolism-related signatures were obtained from previous research (Rosario et al., 2018); the GSVA R package and GSEABase were used to investigate metabolism characteristics of 113 metabolism-related signatures. Consequently, 113 metabolic signatures corresponded to 120 scores within each sample. Specific metabolism-related process signatures in the corresponding subtypes were defined by comparing 113 metabolic scores across all the subtypes using the Limma R package. The Kruskal-Wallis test was used to compare the scores of metabolic scores between subtypes.

### Immune infiltration of LUAD classification

A total of five bioinformatics algorithms, including microenvironment cell population-counter (MCPcounter) (Becht et al., 2016), TIMER (Li et al., 2017), ESTIMATE (Yoshihara et al., 2013), CIBERSORT (Newman et al., 2015), and ssGSEA (Charoentong et al., 2017) were used to investigate immune infiltration characteristic of LUAD classification based on metabolism-related genes. Notably, MCPcounter enables robust quantitation of eight immune cell populations (T cells, CD8 T cells, cytotoxic lymphocytes, B lineage, NK cells, monocytic lineage, myeloid dendritic cells, and neutrophils) in heterogeneous tissues from transcriptome data. TIMER is a component analysis software for tumor-infiltrating immune cells, supporting the analysis of six types of immune cells, including B cell, T cell CD4+, T cell CD8+, neutrophil, macrophage, and myeloid dendritic cell. The ESTIMATE algorithm was used to evaluate stromal and immune cells in malignant tissue based on expression data. Based on the 28 immune cell markers, ssGSEA analysis was conducted to compare the differences in immune scores between different subtypes (Charoentong *et al.*, 2017). Of note, CIBERSORT is an effective tool for deconvolving the expression matrix of human immune cell subtypes based on the linear support vector regression. After five bioinformatics algorithms on metabolism-related genes of LUAD classification, the Kruskal-Wallis test was performed to compare the scores of immune cell infiltration between subtypes. 

### Association of LUAD classification with mutation

MafTools R package was used to analyze and visualize the mutation data between molecular classification. Genes with the highest mutation frequency in LUAD were selected, and a chi-square test was used to confirm the mutation distribution across different molecular classifications. Meanwhile, we calculated the tumor mutation burden (TMB) of each sample and compared the distribution difference of TMB in molecular subtypes.

### Classifier generation based on metabolism-related genes

The differentially expressed metabolism-related genes with absolute FC>1.5 and FDR<0.05 were defined as statistically significant. Then, the significant differentially expressed genes across all the classifications were used to construct the classifier. The top 20 genes within each subtype (only genes with FC>1 were selected) were achieved for the construction of the classifier. The nearest template prediction (NTP) algorithm was used for subclass predictions based on significant differentially expressed genes on GSEdat; the predicted results were compared with the above TCGA LUAD samples classification via ConsensusClusterplus R package. 

### Prediction of immune and targeted therapy on LUAD classification

Existing data on immunotherapy (programmed cell death protein-1 (PD-1) immune checkpoint inhibitor or cytotoxic T-lymphocyte-associated protein-4 (CTLA-4)) for 47 melanoma patients were indirectly used to predict the efficacy of our classification of immunotherapy (Roh et al., 2017). A submap algorithm was used to identify whether the immune therapy of our classification based on metabolism-related genes was similar to melanoma patients. Besides, the pRRophetic R package was applied to value IC50 sensitivity of targeted drugs (bexarotene, doxorubicin, embelin, etoposide, gemcitabine, mitomycin C, vinorelbine, and cisplatin) in molecular classification.

### Statistical analysis

Survival analysis was conducted using the Kaplan-Meier (KM) method, and a log-rank test was used for comparison. Chi-square analysis was used to evaluate the relationship between LUAD classification and clinical features. Unpaired Student’s t-test and Mann-Whitney U-test were used to comparing two groups with normally distributed variables and non-normally distributed variables, respectively. One-way analysis of variance of parametric and Kruskal-Wallis tests of nonparametric variance were used to compare the three groups. Univariate COX and multivariable Cox regression were conducted to evaluate the molecular clusters and classifier using the clinical signatures as concomitant variable (Table S2). All analyses were conducted using R software (version 3.5.1) and SPSS software (version 24). A two-tailed *p*-value <0.05 was considered statistically significant. 

## Results

### Molecular classification of LUAD based on metabolism-related genes

A total of six datasets (GSE19188, GSE29013, GSE30219, GSE31210, GSE37745, GSE50081) from the GEO database were subjected to PCA analysis. Consequently, the results revealed that data differentiation was significantly removed after batch effect, indicating no difference between datasets (Figure S2). After MAD and univariate Cox analyses, 2,752 previously reported metabolism-related genes involved in all metabolic processes and 500 LUAD samples from TCGA were used to classified analysis through ConsensusClusterPlus; *k*= 3 was set as the optimal value of clusters (C1, C2, and C3). When *k*= 3, the consensus matrix heatmap still maintained a clear and sharp boundary, indicating a stable and robust clustering of samples.

**Figure 1 -:**
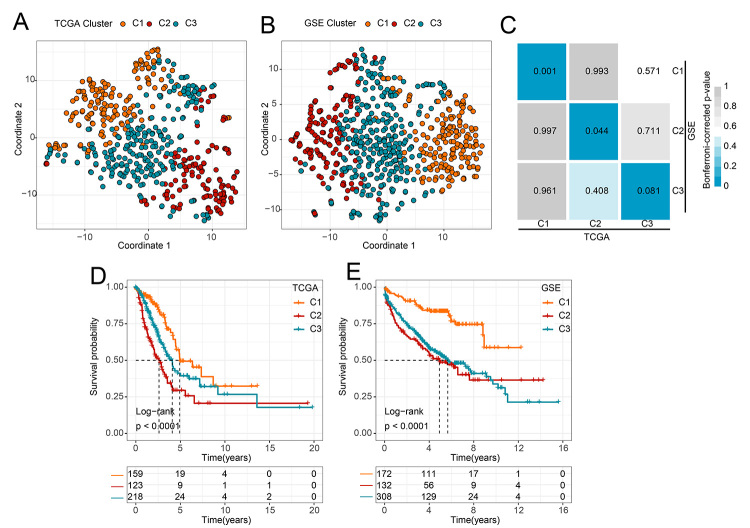
Molecular classification of LUAD based on metabolism-related genes. T-SNE analysis verified the classification of (A) TCGA and (B) GSEdat into 3 clusters. (C) Submap algorithm showing a close association between TCGA and GSEdat. KM analysis to determine the over survival of the 3 clusters in (D) TCGA and (E) GSEdat.

T-SNE methods were used to reduce the dimension of the feature and supported the stratification of three clusters in LUAD samples (Figure 1A). Analyses of molecular classification on GSEdat were similar to the TCGA database (Figure 1B), further verifying the accuracy of three clusters in LUAD samples. Additionally, we performed Submap to confirm the relationship across the three clusters between TCGA and GSEdat. The results showed that C1, C2, and C3 in the TCGA were closely related to the corresponding clusters in GSEdat (Figure 1C). Then, the LUAD samples across the three clusters were analyzed by the KM method, with the best survival for C1 and worst survival for C2 in TCGA (Figure 1D). The KM analysis was validated in GSEdat, and showed that C1 with best survival and C2 with worst survival, although C2 and C3 were indistinguishable with no statistically significance (P=0.42) in terms of patients’ prognosis (Figure 1E, Figure S3A), whiles the survival of C2 was better than C3, and the results were verified in the TCGA (P= 0.0013) (Figure S3B). Generally, C1 has the best prognosis and C2 has the worst prognosis. It could be further used for molecular typing analysis of LUAD based on metabolism.

### Relationship analysis between three clusters and clinical characteristics

**Figure 2 -:**
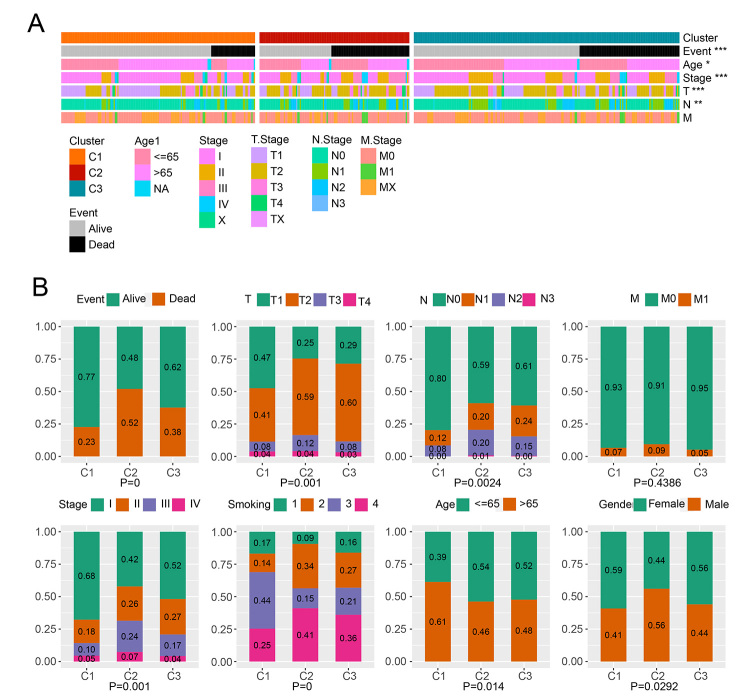
Association of clinical characteristics with the 3 clusters. (A) A cluster map showing association the of 3 clusters with clinical characteristics. (B) Comparison of distribution of clinical characteristics of different molecular subtypes. * *p* < 0.05, **p < 0.01, *** *p* < 0.001, **** *p* < 0.0001.

The clinical characteristic, including living status, topography-lymph node- metastasis (TNM) grade, stage, smoking, age, and gender, were associated with three clusters; the results revealed that the distribution of clinical information in the three clusters displayed a significant difference (Figure 2A). The Chi-square test demonstrated that several clinical characteristics, including alive status, T, N, and stage, were significantly related to three clusters (Figure 2B). C1 was primarily enriched in alive status, T1, N0, and stage Ⅰ indicating a favorable living condition and low grade of the tumor; on the other hand, C2 correlated with dead status, T2 and T3, N1 and N2, as well as stage Ⅱ and Ⅲ; this indicates an unfavorable living condition and relatively high grade of the tumor. Furthermore, the survival differences of molecular clusters were independent of clinical factors, including T, N, and stage (Table S3). Therefore, the above findings indirectly demonstrate good survival of C1 and poor survival of C2.

### Functional analysis of the three clusters

The biological function of the three clusters was analyzed to understand their molecular characteristics further. The metabolism-related genes, significantly different across all the clusters compared to FDR < 0.05 and an absolute value of FC>1.5, were considered for subtype-specific genes. A total of 1,869, 2,203, and 236 specific genes were identified for C1, C2, and C3, respectively. Then, clusterprofiler R package was used to analyze the biological function of KEGG of these specific genes on the three clusters. Consequently, the up-regulated genes of C1 were mainly enriched in the metabolic pathway, while the down-regulated genes were associated with a tumor-related pathway (Figure S4A). In contrast with C1, the enriched genes of C2 were reverse, with up-regulated genes primarily participating in tumor-related pathways while down-regulated genes were related to metabolic pathways (Figure S4B). These results suggest that C1 correlated with high metabolic biological process hence had a good survival. Nevertheless, C2 was associated with tumor-related pathways, indicating carcinogenesis with poor survival. The intermediate survival of C3 engaged in both tumor-related and metabolic pathways in up-and down-regulated genes (Figure S4C).

### Metabolic process signature of the three clusters

**Figure 3 -:**
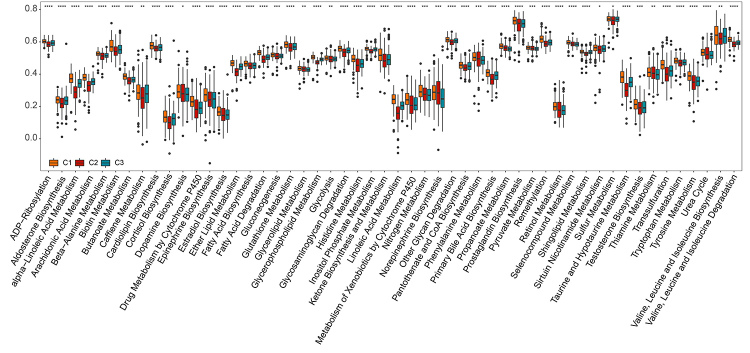
Comparison of metabolic processes across the 3 clusters. The scores of metabolic processes were higher in C1 than in C2 and C3.

The molecular classification of LUAD samples was based on metabolism-related genes; thus, various metabolic processes signatures were investigated across the three clusters. Using the GSVA R package, ssGSEA quantification was performed on 113 metabolic processes on the TCGA dataset. Then, the metabolic process scores between the different subtypes were compared (Figure S5); as a consequence, C1 had the greatest number (51) of metabolic processes, among them, drug metabolism by cytochrome P450, alpha−linoleic acid metabolism, and glutathione metabolism (Figure 3). C2 and C3 had 35 and 10 specific metabolism signatures, respectively (Figure S6A, B). The results reveal that C1 exhibited the highest metabolic activity consistent with KEGG analysis, thereby indicating that C1 was associated with the active metabolic process. To further analyze the characteristic of the clusters, 19 biological features related to tumorigenesis were selected and quantified using the GS VA algorithm (Mariathasan et al., 2018). As a consequence, C1 had a lower score of biological features related to tumorigenesis than that of C2 and C3 (Figure S7).

### Immune infiltration of the clusters

**Figure 4 -:**
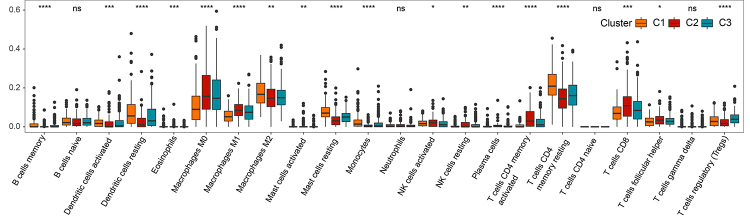
Comparison of immune infiltration scores among the 3 clusters. Immune infiltration scores of the 3 clusters estimated using MCPcounter.

The pathogenesis of tumorigenesis is caused by dysregulation of the immune system (Disis, 2010). Thus, the immune infiltration and immune checkpoint across the clusters were further analyzed to depict the immune landscape. In total, five immune score algorithms, including MCPcounter, TIMER, ESTIMATE, CIBERSORT, and ssGSEA, were used to assess the immune infiltration on the three clusters. Then, the differences in the scores of different immune cells in the three clusters were compared. We found that the immune infiltration score of C1 was relatively higher than that of C2 and C3; C2 had a relatively lower immune infiltration score than C1 and C3 (Figure S8A,). Importantly, the evaluation outcomes of the immune infiltration score were consistent in all five algorithms (Figure 4, Figure S9A-D). Besides, immune checkpoint-related genes were identified from previous studies (Danilova et al., 2019), and the expression of these immune checkpoint genes were compared in the three clusters. Consequently, C1 exhibited the highest expression of immune checkpoint genes, while C2 represented the lowest expression (Figure S8B). All the results indicated that C1 showed robust immunoactivity, high immune infiltration activity, and immune checkpoint function in LUAD. 

**Figure 5 -:**
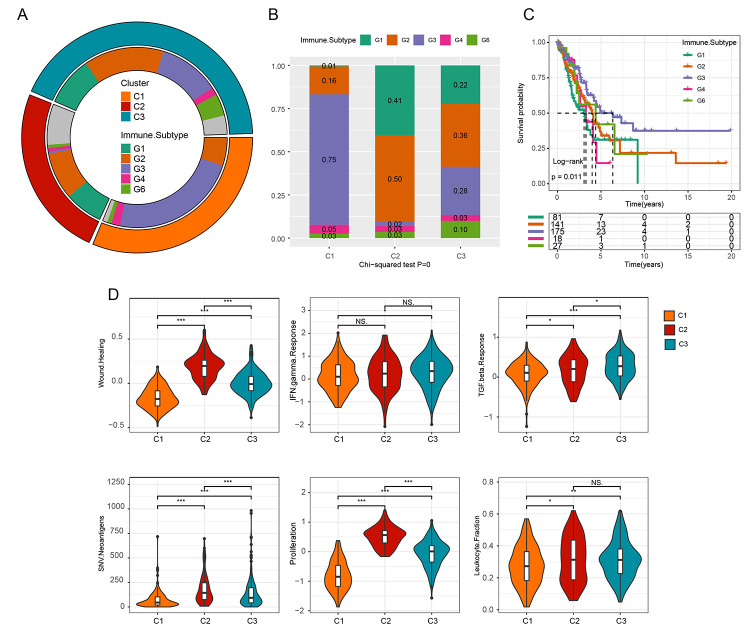
Comparison of 3 clusters with existing immune molecular subtypes in terms of metabolism-related genes. (A) Cyclic graph showing the comparison between 3 clusters and existing immune molecular subtypes. The outer layer shows our molecular subtype, the inner layer displays the existing immunotyping, and the inner gray shows samples without the existing immunotypes and no classification. (B) Analysis of the distribution of existing molecular subtypes among the 3 clusters. (C) KM analysis of the over survival of 6 existing molecular subtypes. (D) the expression of 6 tumor-related signatures in the 3 clusters. * *p* < 0.05, ** *p* < 0.01, *** *p* < 0.001.

Further, six immune subtypes, including G1 (wound Healing), G2 (IFN-γ dominant), G3 (inflammatory), G4 (lymphocyte Depleted), G5 (immunologically quiet), and G6 (TGF-β dominant) in cancer proposed by Thorsson et al. (2018), were used to characterize intratumoral immune states and identify modules of immune signature (Thorsson *et al.*, 2018). The three clusters were compared with six immune subtypes to confirm the accuracy of molecular classification of LUAD based metabolism-related genes. As a result, the distribution of six immune subtypes across the three clusters was significantly different (Figure 5A); C1 primarily comprised G3, while G1 and G2 were mainly distributed in C2 and C3. Kaplan Meier analysis revealed that G3 had the best prognosis, and G1 and G3 were intermediate prognostic (Figure 5B). G3 represented good survival; this was in line with the result of our research, where C1 with a large composition of G3 had the best prognosis in our molecular classification, confirming the accuracy of our molecular classification on LUAD patients. Besides, the six tumor-related signatures (wound healing, IFN gamma response, TGF beta response, proliferation, leukocyte fraction, and SNV neoantigens) (Thorsson *et al.*, 2018) were compared among the three clusters. Although there was no statistical difference, all expressions of the six signatures in C1 were lower than those in C2 and C3 (Figure 5D). 

### Genetic mutation of 3 clusters

**Figure 6 -:**
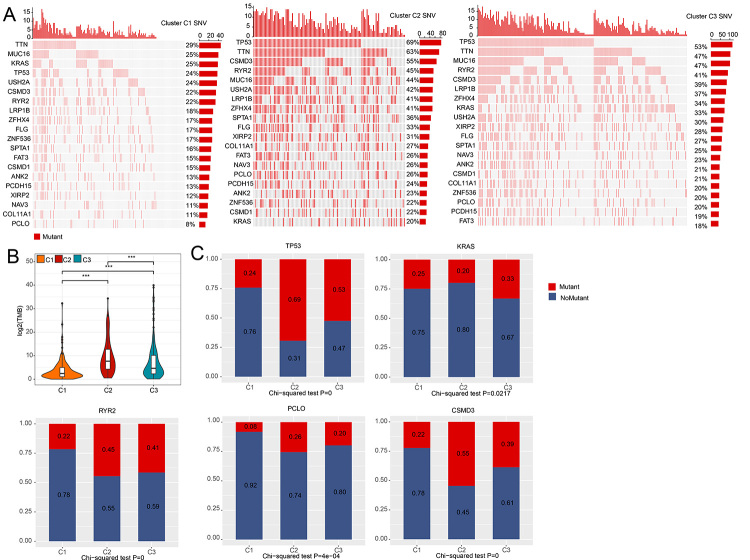
Comparison of the gene mutations among the 3 clusters. (A) Distribution of mutation frequency among the top 20 genes in the 3 clusters. (B) Comparison of gene mutations by TMB among the 3 clusters. (C) Comparison of frequency of mutant genes in the 3 clusters. *** *p* < 0.001.

Somatic variants of genetic mutation is a vital pathogenic mechanism of tumors; thus, the differences in frequencies of somatic variants were analyzed across the three clusters. MafTools R package was used to analyze and visualize the mutation data between molecular classification. A total of 20 genes with the highest frequency for mutation visualization and the genetic mutation across different clusters were diverse, and each cluster of highly mutated genes was also different (Figure 6A). TMB analysis found that there were significant differences in TMB among the 3 clusters; especially, gene mutation frequency in C1 was generally less than that of C2 and C3 (Figure 6B). A total of five mutation genes (TP53 (Skoulidis and Heymach, 2019), KRAS (Ferrer et al., 2018), RYR2 (Yu et al., 2015), PCLO (Qiu et al., 2019), CSMD3 (Zhang et al., 2019)) linked to the pathogenesis of lung cancer were selected to make a comparison between the three clusters. Generally, the mutation frequency of the five genes in C1 was lower than that in C2 and C3 (Figure 6C). Since KRAS mutation and other gene co-occurring mutations have poor effect of immunotherapy and poor prognosis; thus, we further analyzed the mutation and survival of KRAS in LUAD patients. As predicted, the mutation probability of KRAS in LUAD patients is higher than that of non-mutation (Figure S10A), and the prognosis of highly expressed KRAS in LUAD patients was poor although there was no statistical significance (Figure S10B). The above findings suggested that the molecular classification was associated with Genetic mutation and indicated the relationship with clinical prognosis. 

### Generation of classifier

**Figure 7 -:**
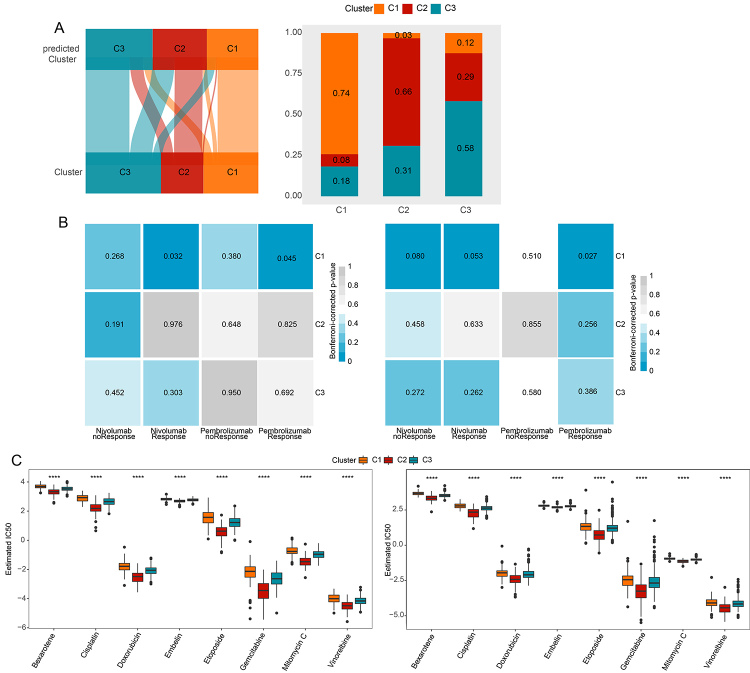
Development of a classifier and comparison of immune and targeted therapy among the 3 clusters. (A) Left: Classification consistency between the 60 genes-classifier and the original prediction based on ConsensusClusterPlus. Right: The distribution between 60 genes-classifier and original prediction based on ConsensusClusterPlus. (B) Sensitivity analysis for responses to nivolumab and pembrolizumab in (left) TCGA and (right) GSEdat databases. (C) Sensitivity of IC50 for targeted drugs in the 3 clusters in (left) TCGA and (right) GSEdat databases. **** *p* < 0.0001.

Several differentially expressed metabolism-related genes were noted in the three clusters; specific informative cluster-related genes signature is necessary to construct the clinical classifier for potential utility. Considering the gene accuracy and clinical application potential, the top 20 genes with FC value > 1 in each cluster were selected to develop the classifier. Thus, a classifier with 60 genes (Figure S11) was generated. Then, the classified prediction of GSEdat was repeated using the 60 gene classifier, and the agreement with the original prediction based on ConsensusClusterPlus was evaluated. The findings revealed that the consistency of the C1, C2, and C3 were 74%, 66%, and 58% (Figure 7A), suggesting the accuracy and repeatability of this 60 genes classifier. Furthermore, univariate COX regression analysis revealed that 60 genes classifier were independent of T, N, M, and stage (Table S4); and 60 genes classifier was an independent clinical factor by multivariable Cox regression analysis (Table S5).

### Sensitivity of immune and targeted therapy on the clusters

The above results revealed distinctly diverse immune infiltration and checkpoint across the three clusters; we sought to understand whether the immune and targeted therapy between the three clusters was different. The expression profiles of the clusters (C1, C2, and C3) were compared with that of another published dataset (Roh et al., 2017) that included patients treated with nivolumab and pembrolizumab. Consequently, a significant relationship between C1 and nivolumab and pembrolizumab response was observed in the TCGA database (Figure 7B). Besides, C1 correlated with pembrolizumab response in the GSEdat database (Figure 7B). A comparison of the sensitivity of therapeutic targets was conducted across the clusters. In TCGA and GSEdat databases, the sensitivity of therapeutic targets in C1 was higher than that in C2 and C3 (Figure 7C); this indicates the high efficacy of therapeutic targets for C1. These findings indicate that the LUAD samples of C1 have good sensitivity and response of immune and targeted therapy, thereby indirectly suggesting a good survival of LUAD samples in C1.

## Discussion

Molecular classification of LUAD tumors has been proposed in recent years, including subtypes based on immune infiltration, proteomics, non-coding RNA, and epigenetic modification (Borczuk, 2016; Chalela et al., 2017). Nevertheless, classification based on metabolism-related gene features has not been adequately investigated. Considering that tumorigenesis is a metabolic reprogramming process and that dysregulation of metabolic genes is vital for regulating tumorigenesis (Carrer and Wellen, 2015; Li and Zhang, 2016), we classified LUAD with metabolic genes to improve the understanding of molecular characteristics and specific classification of LUAD. Specifically, an integrative classification of the metabolism profile of LUAD samples was performed to divide the patients into three clusters. Then, the clinical features, metabolic signatures, immune infiltration, gene mutation, immunotherapy, and targeted drugs were used to associate the clusters. The focus of this study was molecular typing based on metabolic genes. The data information provided may complement the pathological typing to obtain information that was helpful for clinical diagnosis and treatment. Of course, our research was still preliminary and exploratory, the research data and information provided in this study may have reference value in the future. It was of certain significance to study the molecular typing of complementary metabolic genes in LUAD. We suggested that combining our molecular typing with the existing molecular typing (immunity, noncoding RNA, gene, etc.) has certain value for the precise treatment of LUAD.

Consequently, all the clusters exhibited different molecular characteristics, i.e., C1 correlated with good prognostic, high metabolic activity, high immune response, low gene mutation frequency, high immunotherapy sensitivity, and targeted therapy. Meanwhile, C2 had a poor prognosis, displaying low metabolic activity, low immune response, high frequency of gene mutation, low sensitivity of immunotherapy, and targeted therapy which significantly conflicted C1 clusters. We systematically classified LUAD based on metabolism-related genes and comprehensively depicted molecular characteristics of subtypes, which promoted the understanding of clinical individualization and precise therapy on LUAD patients. At present, some molecular typing has been applied in clinic and has brought great benefits to patients (Cooper et al., 2013). There have been relevant studies on molecular analysis of lung cancer (immunity, non-coding RNA, genes, etc.) (Sun et al., 2020), which has promoted the development of diagnosis and treatment of lung cancer. The biggest difference from their research was that this study was located in the metabolic field closely related to the pathogenesis of cancer. We used metabolic genes to carry out molecular typing of LUAD, which was rarely studied. Because the occurrence of lung cancer is closely related to metabolism (Stocke et al., 2018), it may be of great significance to study the molecular typing based on lung cancer metabolic genes. My research may be combined with their existing achievements to provide more abundant research reference data.

The metabolic characteristics of LUAD classification showed that C1 is associated with numerous metabolic processes and functional analysis revealed that up-regulated genes of C1 mainly participated in the metabolic pathway. Cell metabolic changes promote transformation and tumor progression; therefore, metabolic phenotypes of cancer can be used for tumor imaging, thereby providing prognostic information and cancer treatment (Vander Heiden and DeBerardinis, 2017). Cancer cells reprogram their metabolism to meet the biosynthetic needs of tumor growth and proliferation; besides, different metabolic pathways have different roles, including metabolic enhancement and inhibition (Luengo et al., 2017). Choi and Na (2018) discovered that the metabolic landscape across all cancers reveals prognostic pathways, poor prognostic metabolic signatures, including pyrimidine and purine metabolism, whereas good prognostic signatures include lipid and fatty acid oxidation metabolism (Choi and Na, 2018); this was according to the abundant lipid and fatty metabolism in C1 with good prognostic and adequate pyrimidine and purine metabolism in C2 with poor prognostic. Wang et al. (2019) revealed that lower UDP-glucose metabolism was closely associated with metastasis and recurrence of lung cancer; this indicates that lower UDP-glucose metabolism implies poor prognosis (Wang *et al.*, 2019). Herein, the glycolysis-related metabolism process was enriched in C1 with a good prognosis. The development of tumors through high metabolism has been reported; however, specific metabolic activities can directly be implicated in transformation processes or inhibit biological processes of tumor growth (Vander Heiden and DeBerardinis, 2017); this may be the reason for the favorable prognosis of tumors with hypermetabolism. Further, we found that the C1 hypermetabolism group had a good prognosis, thereby providing a basis for further research on tumor pathogenesis. Importantly, for the sake of research integrity and repeatability, we have set up two datasets, of which GEO also included six subsets. Whether it was the accuracy of molecular typing of gene metabolism genes or the correlation between molecular typing and clinical information, the comparative analysis between the experimental group and the validation was set up, which could more accurately show the accuracy and repeatability of our experiment.

Recent efforts against cancer primarily focus on strengthening immune activation mechanisms to promote immune activation by regulating immune regulation and immune activation mechanisms (Fujii and Shimizu, 2019). This work found that C1 with a good prognosis exhibited robust immunoactivity. We conducted five algorithms (MCPcounter, TIMER, ESTIMATE, CIBERSORT, and ssGSEA) for detection to eliminate analysis errors and obtained similar outcomes, i.e., C1 had a high immune score. Based on previous research, lung cancer patients at low risk were strongly enriched for genes associated with immune response (Roepman et al., 2009). Nevertheless, systemic immune activation did not necessarily trigger cancer regression, specifically in treating patients with solid tumors. Since cancer cells did not progress fast to fight the immune response, they used various strategies to escape (Sanmamed and Chen, 2018). Novel approaches for the immune response to cancer, including programmed death receptor 1 (PD-1) or its ligand (PD-L1), should be included to block these immune escape mechanisms (Yi et al., 2018). Therefore, we also estimated the gene scores associated with the immune checkpoint and found that C1 was the highest cluster. CD40LG, with the highest expression in C1, potentially induces an immune response to kill tumor cells by recruiting and activating the enhanced immune effectors to overcome immune escape (Kuhn et al., 2019). Our study focuses on the immune system as a prognostic marker and novel target therapy for LUAD based on metabolism-related genes classification.

Regarding gene mutation, C1 with a good prognosis and C2 with a poor prognosis demonstrated a low and high frequency of gene mutation, respectively. Gene mutation in cancer cells provides a means for overexpressing cancer-promoting driver genes or suppressing anti-oncogene (Roepman et al., 2009). Several common tumor-related genes, including *TP53* and *KRAS,* had lower expression in C1 than in the other two clusters. *TP53* gene is a human tumor suppressor gene; its variants are associated with lung cancer risk, prognosis, and somatic mutations in lung tumors (Mechanic et al., 2007). C2 demonstrated high expression of *TP53* mutation, thus exhibiting high risk and poor prognosis. Specifically, reports indicate that lung cancer with dual mutations in *TP53* and other genes may have a worse prognosis (Hata et al., 2010). To our expectation, other common tumor-related genes, including *KRAS*, *RYR2*, *PCLO*, and *CSMD3,* had a lower expression in C1 than that in C2. We hypothesized that *TP53* mutation could function with mutations in other genes to increase anti-cancer resistance, representing a poor prognosis. Besides, we evaluated the sensitivity of immune and targeted therapy on the three clusters. C1 was associated with immune therapy and was related to targeted therapy, implying a good prognosis of C1. High metabolism, robust immune activity, and low genes mutations of C1 may benefit from immune and targeted therapy; this suggests a favorable prognosis.

## Conclusion

We classified LUAD into three clusters based on metabolism-related genes and characterized them with molecular characteristics. Consequently, the three clusters exhibited distinct features in metabolic signatures, immune processes and checkpoint, genetic mutation, sensitive drug response, and clinical characteristics. Our findings provide valuable information to improve the understanding of the molecular characteristics of LUAD and may help to improve the clinical management of LUAD. 

## Supplementary material

The following online material is available for this article:

Figure S1 - A flow chart of the study.

Figure S2 - PCA analysis for the GSEdat.

Figure S3 - KM analysis between C2 and C3 in (A) GSEdat and (B) TCGA databases.

Figure S4 - The biological functions for the 3 clusters.

Figure S5 - Heatmap showing ssGSEA scores of the molecular subtypes of various metabolic processes.

Figure S6 - Comparison of metabolic processes across the 3 clusters.

Figure S7 - Comparison of scores of 19 biological features related tumorigenesis for the 3 clusters.

Figure S8 - Immune infiltration scores of each molecular subtype.

Figure S9 - Comparison of immune infiltration scores among the 3 clusters.

Figure S10 - Mutation and survival of KRAS in LUAD patients.

Figure S11 - The top 20 genes in the 3 clusters used to construct the classifier.

Table S1 - The clinical information of TCGA and GSEdat databases.

Table S2 - 408 metabolism-related genes were obtained for molecular typing.

Table S3 - The association between molecular clusters and clinical signatures by multivariable Cox regression analysis.

Table S4 - The association between 60 genes classifier and clinical signatures by univariate Cox regression analysis.

Table S5 - The association between 60 genes classifier and clinical signatures by multivariable Cox regression analysis.
