# Supplementary material for: Systematically analyzed molecular characteristics of lung adenocarcinoma using metabolism-related genes classification
Source: Genet Mol Biol. 2023 Jan 6;45(4):e20220121. doi: 10.1590/1678-4685-GMB-2022-0121 (PMC9830935; doi:10.1590/1678-4685-GMB-2022-0121)
Supplement: Figure S1 - [file 1415-4757-GMB-45-4-e20220121-s1.pdf]

Genetics and Molecular Biology

https://doi.org/ 10.1590/1678-4685-GMB-2022-0121

Supplementary Material to "Systematically analyzed molecular characteristics of lung adenocarcinoma using metabolism-related genes classification"

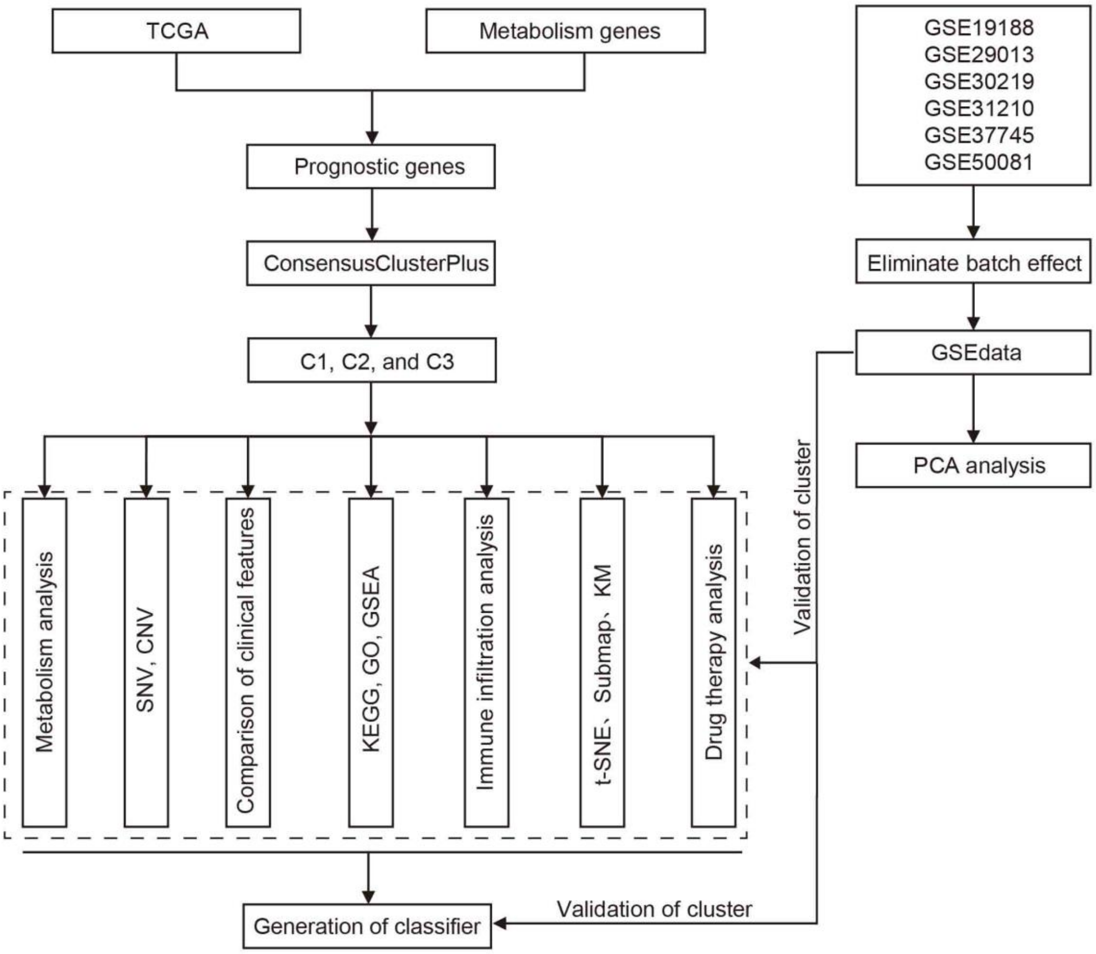

Figure S1. A flow chart of the study.
