# Supplementary material for: Systematically analyzed molecular characteristics of lung adenocarcinoma using metabolism-related genes classification
Source: Genet Mol Biol. 2023 Jan 6;45(4):e20220121. doi: 10.1590/1678-4685-GMB-2022-0121 (PMC9830935; doi:10.1590/1678-4685-GMB-2022-0121)
Supplement: Figure S2 - [file 1415-4757-GMB-45-4-e20220121-s2.pdf]

Genetics and Molecular Biology

https://doi.org/ 10.1590/1678-4685-GMB-2022-0121

Supplementary Material to "Systematically analyzed molecular characteristics of lung adenocarcinoma using metabolism-related genes classification"

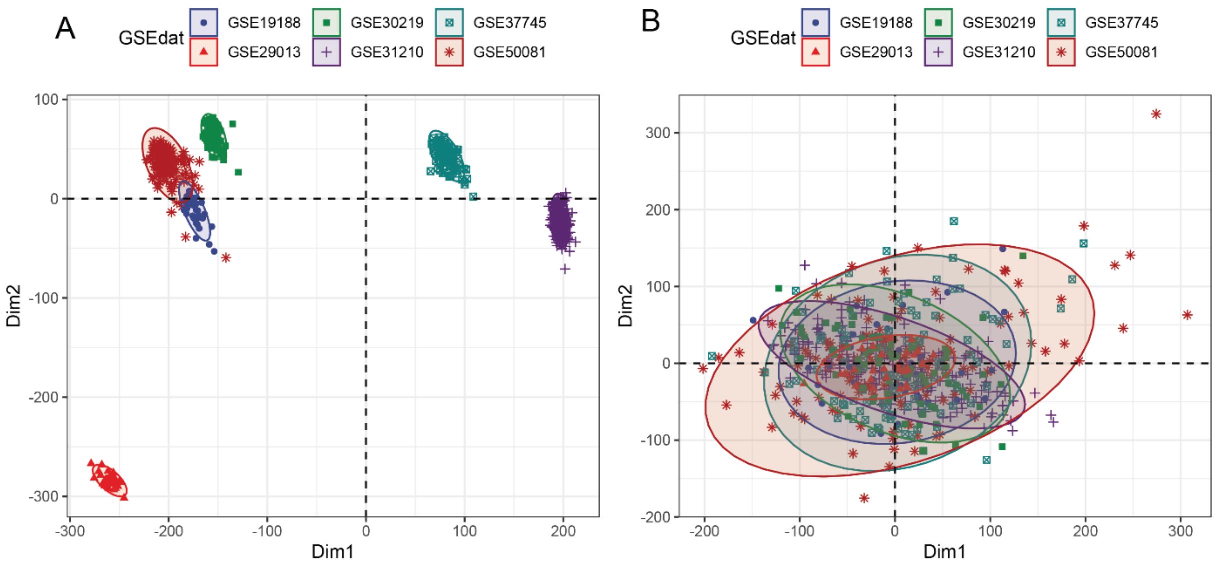

Figure S2. PCA analysis for the GSEdat. (A) PCA analysis for the six GSE datasets before elimination of batch effect. (B) PCA analysis for the six GSE datasets after elimination of batch effect.
