# Supplementary material for: Systematically analyzed molecular characteristics of lung adenocarcinoma using metabolism-related genes classification
Source: Genet Mol Biol. 2023 Jan 6;45(4):e20220121. doi: 10.1590/1678-4685-GMB-2022-0121 (PMC9830935; doi:10.1590/1678-4685-GMB-2022-0121)
Supplement: Figure S3 - [file 1415-4757-GMB-45-4-e20220121-s3.pdf]

Genetics and Molecular Biology

https://doi.org/ 10.1590/1678-4685-GMB-2022-0121

Supplementary Material to "Systematically analyzed molecular characteristics of lung adenocarcinoma using metabolism-related genes classification"

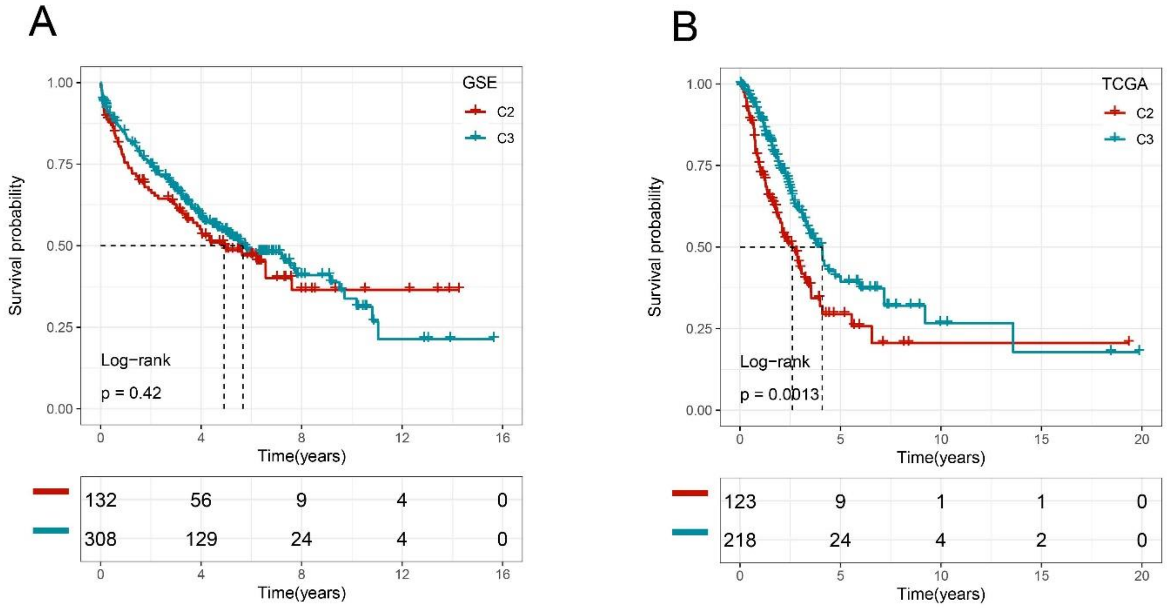

Figure S3. KM analysis between C2 and C3 in (A) GSEdat and (B) TCGA databases.
