# Supplementary material for: Systematically analyzed molecular characteristics of lung adenocarcinoma using metabolism-related genes classification
Source: Genet Mol Biol. 2023 Jan 6;45(4):e20220121. doi: 10.1590/1678-4685-GMB-2022-0121 (PMC9830935; doi:10.1590/1678-4685-GMB-2022-0121)
Supplement: Figure S6 - [file 1415-4757-GMB-45-4-e20220121-s6.pdf]

Genetics and Molecular Biology

https://doi.org/ 10.1590/1678-4685-GMB-2022-0121

Supplementary Material to "Systematically analyzed molecular characteristics of lung adenocarcinoma using metabolism-related genes classification"

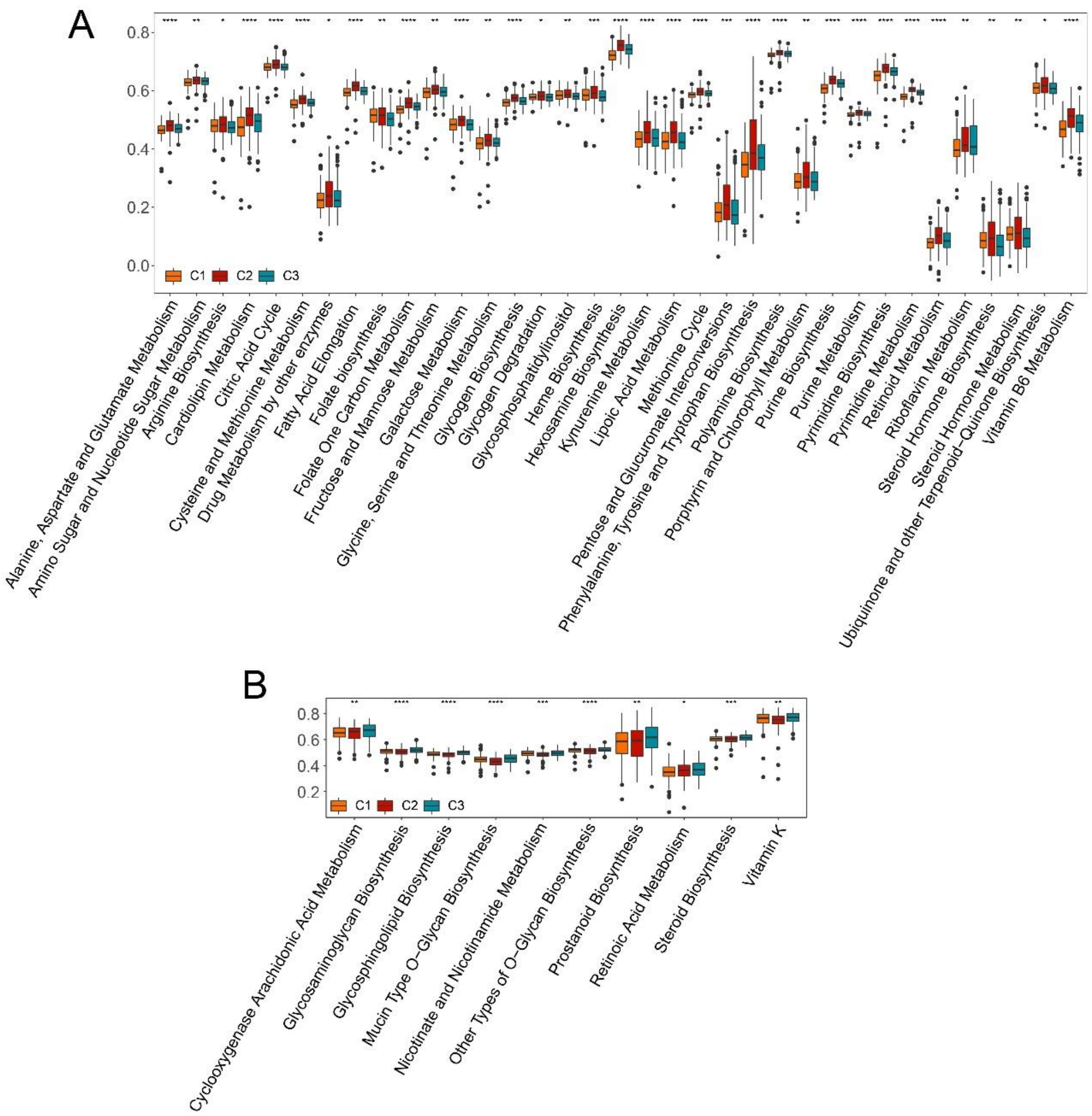

Figure S6. Comparison of metabolic processes across the 3 clusters. (B) The scores of metabolic processes were higher in C2 than in C1 and C3. (C) The scores of metabolic processes were higher in C3 than in C1 and C2. *P < 0.05, **P < 0.01, ***P < 0.001, ****P < 0.0001.
