# Supplementary material for: Systematically analyzed molecular characteristics of lung adenocarcinoma using metabolism-related genes classification
Source: Genet Mol Biol. 2023 Jan 6;45(4):e20220121. doi: 10.1590/1678-4685-GMB-2022-0121 (PMC9830935; doi:10.1590/1678-4685-GMB-2022-0121)
Supplement: Figure S8 - [file 1415-4757-GMB-45-4-e20220121-s8.pdf]

Genetics and Molecular Biology

https://doi.org/ 10.1590/1678-4685-GMB-2022-0121

Supplementary Material to "Systematically analyzed molecular characteristics of lung adenocarcinoma using metabolism-related genes classification"

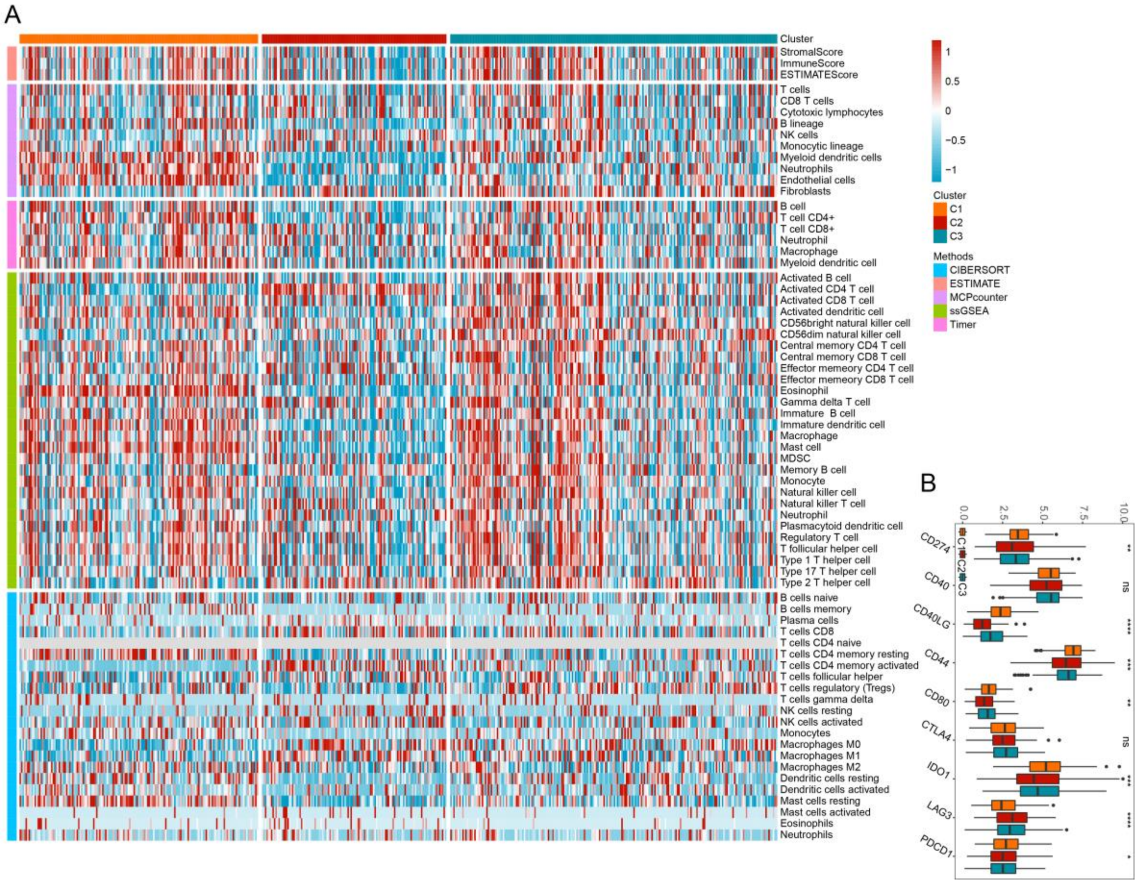

Figure S8. Immune infiltration scores of each molecular subtype. (A) Heat map of immune cell infiltration scores for the molecular subtypes as determined using the five immune assessment software. (B) Expression of immune checkpoints in various molecular subtypes. ** p < 0.01, *** p < 0.001, **** p < 0.0001.
