# Supplementary material for: Systematically analyzed molecular characteristics of lung adenocarcinoma using metabolism-related genes classification
Source: Genet Mol Biol. 2023 Jan 6;45(4):e20220121. doi: 10.1590/1678-4685-GMB-2022-0121 (PMC9830935; doi:10.1590/1678-4685-GMB-2022-0121)
Supplement: Figure S9 - [file 1415-4757-GMB-45-4-e20220121-s9.pdf]

Genetics and Molecular Biology

https://doi.org/ 10.1590/1678-4685-GMB-2022-0121

# Supplementary Material to "Systematically analyzed molecular characteristics of lung adenocarcinoma using metabolism-related genes classification"

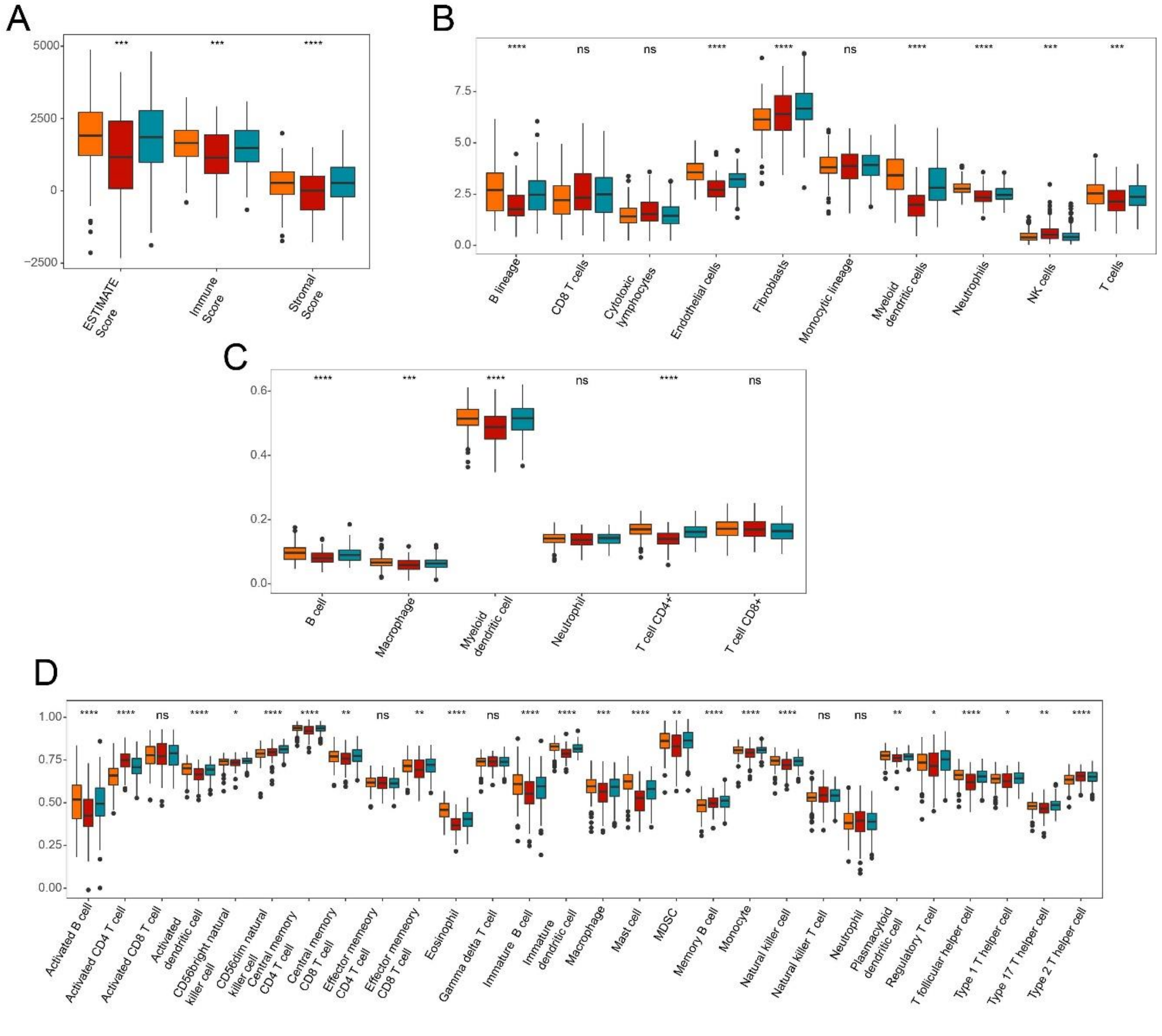


**Figure S9.** Comparison of immune infiltration scores among the 3 clusters. Immune infiltration scores of the 3 clusters estimated using (A) TIMER, (B) ESTIMATE, (C) CIBERSORT, and (D) ssGSEA. * $p < 0.05$, ** $p < 0.01$, *** $p < 0.001$, **** $p < 0.0001$.
