# Supplementary material for: Systematically analyzed molecular characteristics of lung adenocarcinoma using metabolism-related genes classification
Source: Genet Mol Biol. 2023 Jan 6;45(4):e20220121. doi: 10.1590/1678-4685-GMB-2022-0121 (PMC9830935; doi:10.1590/1678-4685-GMB-2022-0121)
Supplement: Figure S10 - [file 1415-4757-GMB-45-4-e20220121-s10.pdf]

Genetics and Molecular Biology

https://doi.org/ 10.1590/1678-4685-GMB-2022-0121

Supplementary Material to "Systematically analyzed molecular characteristics of lung adenocarcinoma using metabolism-related genes classification"

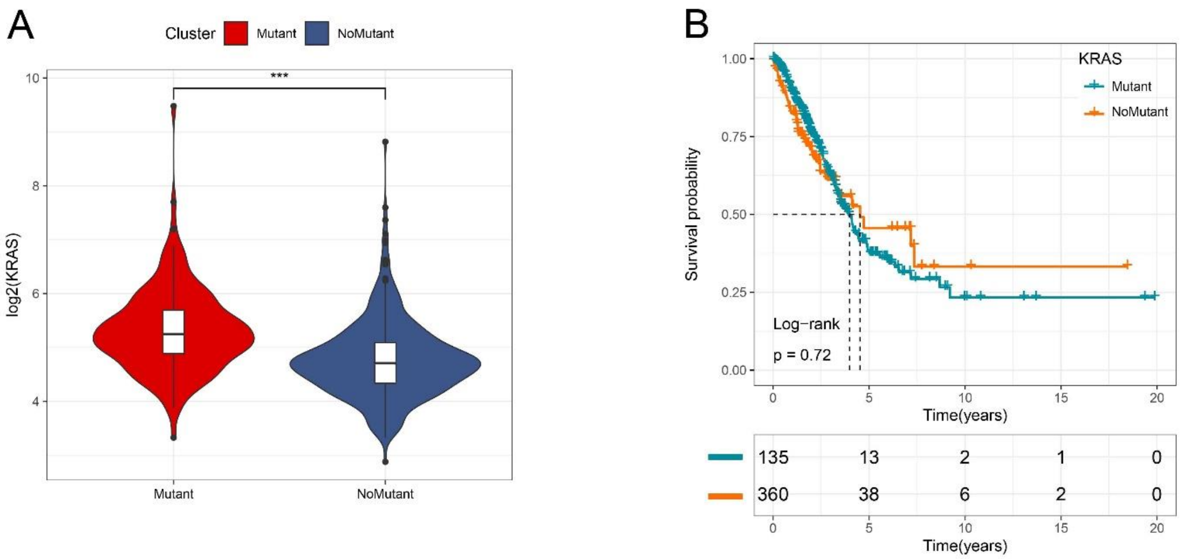

Figure S10. Mutation and survival of KRAS in LUAD patients. (A) The comparison between mutation and non-mutation probability of KRAS across LUAD patients. (B) KM analysis of survival between high and low expression KRAS.
