# Supplementary material for: Systematically analyzed molecular characteristics of lung adenocarcinoma using metabolism-related genes classification
Source: Genet Mol Biol. 2023 Jan 6;45(4):e20220121. doi: 10.1590/1678-4685-GMB-2022-0121 (PMC9830935; doi:10.1590/1678-4685-GMB-2022-0121)
Supplement: Table S1 - [file 1415-4757-GMB-45-4-e20220121-s12.pdf]

Genetics and Molecular Biology
https://doi.org/ 10.1590/1678-4685-GMB-2022-0121

# Supplementary Material to "Systematically analyzed molecular characteristics of lung adenocarcinoma using metabolism-related genes classification"

**Table S1.** The clinical information of TCGA and GSEdat databases.

| Feature | TCGA | GSEdat |
|---|---|---|
| Event | | |
| Alive | 318 | 374 |
| Dead | 182 | 238 |
| Gender | | |
| Female | 270 | |
| Male | 230 | |
| Age | | |
| >65 | 253 | |
| <=65 | 237 | |
| Unknown | 10 | |
| T Stage | | |
| T1 | 167 | |
| T2 | 267 | |
| T3 | 45 | |
| T4 | 18 | |
| TX | 3 | |
| N Stage | | |
| N0 | 324 | |
| N1 | 94 | |
| N2 | 69 | |
| N3 | 2 | |
| NX | 11 | |
| M Stage | | |
| M0 | 332 | |
| M1 | 24 | |
| MX | 144 | |
| Stage | | |
| I | 268 | |
| II | 119 | |
| III | 80 | |
| IV | 25 | |
| X | 8 | |

| **Feature** | **TCGA** | **GSEdat** |
|---|---|---|
| Smoking | | |
| 1 | 71 | |
| 2 | 119 | |
| 3 | 129 | |
| 4 | 163 | |
| 5 | 4 | |
| 7 | 14 | |
