# Supplementary material for: Systematically analyzed molecular characteristics of lung adenocarcinoma using metabolism-related genes classification
Source: Genet Mol Biol. 2023 Jan 6;45(4):e20220121. doi: 10.1590/1678-4685-GMB-2022-0121 (PMC9830935; doi:10.1590/1678-4685-GMB-2022-0121)
Supplement: Table S2 - [file 1415-4757-GMB-45-4-e20220121-s13.pdf]

Genetics and Molecular Biology
https://doi.org/ 10.1590/1678-4685-GMB-2022-0121

Supplementary Material to "Systematically analyzed molecular characteristics of lung adenocarcinoma using metabolism-related genes classification"

Table S2. 408 metabolism-related genes were obtained for molecular typing.

Genes	P.Value	HR	lower.95	upper.95
ST3GAL6	0.002972	0.748226	0.617904	0.906034
PPOX	0.026155	0.777008	0.622094	0.970499
CYP2D6	0.043823	0.854757	0.733791	0.995664
KCNJ11	0.035414	0.872779	0.768854	0.990752
SLC18A2	0.000937	0.708769	0.578029	0.869079
MCOLN2	0.012604	0.814676	0.693508	0.957013
HS3ST3A1	0.019239	1.234172	1.034829	1.471917
SLC20A1	0.02648	1.219254	1.02342	1.452563
PTGR1	0.011795	1.164352	1.034308	1.310745
ABCC1	0.018402	1.23932	1.036838	1.481345
ACAD8	0.00824	0.806651	0.6878	0.946038
ABCF2	0.04317	1.33501	1.008912	1.766508
SLC39A10	0.013518	1.276583	1.051701	1.54955
CHRNA5	0.032765	1.123891	1.009619	1.251096
SRXN1	0.014095	1.154914	1.02946	1.295656
ATP1B3	0.024598	1.271703	1.031243	1.568233
CA4	0.008422	0.839009	0.736302	0.956043
ENO1	0.001319	1.454665	1.157281	1.828465
ATP8B2	0.029117	0.841264	0.720279	0.982572
GANAB	0.004248	1.440231	1.121566	1.849437
SLC7A1	0.033336	1.203359	1.014726	1.427059
FHIT	0.035183	0.753403	0.57889	0.980526
GCLC	0.004773	1.13067	1.038226	1.231346
FBP1	0.002784	0.820109	0.720156	0.933935
ATP11B	0.011207	1.300642	1.06154	1.593599
INPP5B	0.002088	0.71542	0.578005	0.885504
KCTD15	0.006124	1.250401	1.065752	1.467042
IPMK	0.04099	1.262945	1.009603	1.579858
BLVRB	0.006476	1.240143	1.062144	1.447971
ST6GALNAC6	0.0117	0.769298	0.627394	0.943298
HS3ST2	0.003382	0.840258	0.747932	0.943981
B3GNT3	0.003448	1.178386	1.055638	1.315406

Genes	P.Value	HR	lower.95	upper.95
CYC1	0.021928	1.240119	1.031635	1.490735
ENO3	0.045839	0.901282	0.813871	0.998081
HTR3A	0.026678	1.096097	1.010665	1.188751
SGPP1	0.024014	1.234366	1.028091	1.482029
KCTD7	0.022511	0.753395	0.590722	0.960864
ATP1A2	0.013853	0.765538	0.618811	0.947054
SLC25A27	0.045207	0.854712	0.732985	0.996654
ATP2A2	0.039975	1.246314	1.010133	1.537718
GFPT2	0.001982	1.248265	1.084621	1.4366
PIGX	0.008591	1.389938	1.087292	1.776825
SLC6A8	0.004746	1.178046	1.051404	1.319943
ECH1	0.02903	1.264751	1.024295	1.561654
CLIC1	0.000851	1.548388	1.197597	2.001931
SLC25A24	0.03679	1.224282	1.012493	1.480372
PTER	0.010535	1.323805	1.067757	1.641255
DHCR7	0.005758	1.283778	1.075185	1.532839
FLAD1	0.029058	1.303333	1.027404	1.653368
NUDT1	0.000524	1.433492	1.169533	1.757026
SLC25A42	0.000135	0.665802	0.540299	0.820459
CLIC6	0.000113	0.870589	0.811451	0.934036
NME2	0.002708	1.342961	1.107567	1.628383
COX5B	0.016804	1.299295	1.048328	1.610342
LPCAT4	0.01914	1.244153	1.036341	1.493636
UAP1	0.011628	1.349772	1.069248	1.703893
KCNJ14	0.003954	1.417431	1.118052	1.796975
EPHX2	0.032071	0.844781	0.724039	0.985659
CLIC5	0.02581	0.892574	0.807694	0.986374
PLA2G4F	0.008556	0.855369	0.761341	0.96101
CLCN3	0.00881	1.357637	1.080005	1.70664
ATP8A1	0.00041	0.793833	0.698408	0.902297
NT5DC3	0.04082	1.220392	1.00835	1.477023
SULT2B1	0.002281	1.18558	1.062763	1.322591
SCCPDH	0.029679	1.220511	1.019842	1.460665
ABCA5	0.033947	0.800659	0.651948	0.983291
SLCO4A1	0.00102	1.199507	1.076133	1.337025
FMO2	0.007816	0.85517	0.762053	0.959666
ENTPD7	0.026441	1.218848	1.023426	1.451587
HCN2	0.001617	1.279525	1.097744	1.491409
AQP3	0.025056	0.916966	0.850004	0.989203
CYP2U1	0.017898	0.780904	0.636343	0.958305
KCTD5	0.008804	1.33109	1.07467	1.648693
ACAT1	0.047676	0.822202	0.677372	0.997997

Genes	P.Value	HR	lower.95	upper.95
GPAM	0.044844	0.820821	0.676814	0.995468
SLC22A23	0.018683	0.857734	0.754761	0.974756
FMO3	0.002815	0.80483	0.697969	0.928052
FAAH	0.001755	0.769054	0.652407	0.906557
CA3	0.026835	0.86088	0.753958	0.982964
SLC35A2	0.036356	1.236292	1.013567	1.507961
PDE6B	0.037374	0.846098	0.722911	0.990278
CYP27C1	0.036396	1.140966	1.008386	1.290977
AQP4	0.005333	0.912941	0.856281	0.97335
PLA2G3	0.011519	0.83601	0.727557	0.960629
ALDH2	0.010263	0.823756	0.710404	0.955196
SLC16A13	0.00675	1.320068	1.079799	1.613799
PLA2G1B	0.008336	0.904263	0.839122	0.974462
OASL	0.012933	1.161908	1.032244	1.307859
SFXN1	0.000424	1.515129	1.202574	1.908918
PGS1	0.010036	0.724539	0.566931	0.925963
SULT1A1	0.01435	0.817007	0.694962	0.960485
SLC35C1	0.029724	1.237576	1.021185	1.499821
NDUFA12	0.004837	1.482063	1.127234	1.948584
IMPAD1	0.015291	1.304246	1.052296	1.61652
KCNQ1	0.01819	0.884917	0.799537	0.979416
ELOVL3	0.039329	1.143135	1.006562	1.298238
SLC26A5	0.011897	0.707543	0.540346	0.926474
ST3GAL4	0.002251	1.254223	1.084577	1.450404
B4GALNT1	0.006938	1.179056	1.046173	1.328818
ARSG	0.021622	0.710881	0.531296	0.951169
PLD4	0.008036	0.794677	0.670481	0.941879
ADH1B	0.006177	0.901501	0.837013	0.970958
LPL	0.028189	0.911344	0.838833	0.990122
DHFR	0.00139	1.409193	1.141919	1.739026
GRIK2	0.001751	1.334245	1.113789	1.598337
ECHDC2	0.00481	0.766535	0.637183	0.922146
SPR	0.031918	1.258992	1.020109	1.553815
CRYL1	0.023861	0.796601	0.653987	0.970315
PGK1	0.010658	1.25306	1.05385	1.489927
B4GALT6	0.005132	1.234159	1.065078	1.430081
SLC34A2	0.000783	0.894092	0.837552	0.954449
ENPP5	0.000106	0.770124	0.674867	0.878826
FUCA1	0.007037	0.781493	0.653215	0.934963
SLC25A43	0.043172	1.25842	1.007082	1.572483
KCTD11	0.034243	1.308808	1.020188	1.679083
PIP5KL1	0.036223	0.865706	0.756416	0.990785

**Genes**	**P.Value**	**HR**	**lower.95**	**upper.95**
GMPR	0.025539	0.850656	0.738076	0.980408
ATP1B2	0.023452	0.788585	0.642137	0.968432
ACSS3	0.010524	0.780082	0.644913	0.943581
SLC46A3	0.001169	0.767315	0.653927	0.900363
MINPP1	0.023781	1.345691	1.04028	1.740765
CHKA	0.001988	0.760349	0.639128	0.90456
MGAT2	0.028324	1.270443	1.025739	1.573526
MDH2	0.002856	1.468909	1.140949	1.891139
ALOX15	0.032635	0.894992	0.808389	0.990873
GALNS	0.043208	1.269164	1.007305	1.599096
PGM2	0.00028	1.396106	1.166089	1.671493
ACOT7	0.000923	1.383254	1.141672	1.675956
PLCB3	0.004545	1.418413	1.114169	1.805736
SLC4A8	0.000929	0.716065	0.587624	0.872581
SLC2A11	0.014649	0.725931	0.561307	0.938836
SLC44A1	0.000849	1.454659	1.167207	1.812904
LDHD	0.009051	0.857699	0.764321	0.962486
SLC37A1	0.029535	1.273944	1.024357	1.584343
FH	0.028147	1.360926	1.033579	1.791949
TCN1	0.000483	1.094924	1.040557	1.15213
SRM	0.006038	1.404352	1.102053	1.789573
AMY2B	0.032974	0.829844	0.699107	0.985031
CMAS	0.000291	1.433145	1.179638	1.741132
ADSSL1	0.028323	1.165052	1.016352	1.335507
HMBS	0.016146	1.306275	1.050741	1.623953
PIK3CG	0.004638	0.800066	0.685586	0.933662
SLC24A3	0.031585	0.869124	0.764783	0.9877
PTGES	0.030686	1.12233	1.0108	1.246166
SLC25A32	0.03798	1.309325	1.015062	1.688895
GALNT11	0.007763	0.74652	0.601967	0.925785
SLC27A1	0.036394	0.811115	0.666688	0.986828
GSS	0.003795	1.46356	1.130869	1.894126
GPX8	0.000207	1.305696	1.13409	1.503269
ST3GAL5	0.001723	0.833562	0.743873	0.934065
NME1	0.009635	1.275595	1.060898	1.533741
PPAT	0.004462	1.298518	1.084553	1.554695
SLC25A34	0.014129	0.68919	0.511944	0.927803
GRIN2D	0.041197	1.112298	1.004262	1.231956
SOD1	0.002626	1.444393	1.136721	1.835343
IVD	0.002129	0.755025	0.631088	0.9033
NIPA1	0.015707	1.350016	1.058246	1.72223
PYGB	0.003641	1.27105	1.081313	1.49408

Genes	P.Value	HR	lower.95	upper.95
SRR	0.048336	1.32711	1.002071	1.757581
FMO4	0.017638	0.796682	0.660323	0.9612
MTHFS	0.001364	1.460707	1.158348	1.841989
FRRS1	0.000851	1.324317	1.122818	1.561976
INMT	0.001623	0.857068	0.778681	0.943346
ATP8A2	0.000142	0.655769	0.527635	0.815021
P2RX1	0.00066	0.712647	0.586411	0.866058
CA9	0.020927	1.072308	1.010617	1.137765
MOCS1	0.004627	0.764048	0.634203	0.920476
PTDSS1	0.001624	1.51702	1.170666	1.965846
SCNN1B	0.001379	0.871465	0.801017	0.948108
KCNK5	0.020236	0.885413	0.798972	0.981207
MAOA	0.046672	0.904926	0.820096	0.99853
MTHFD2	0.004101	1.248803	1.073008	1.453399
NT5E	0.009185	1.121921	1.028899	1.223354
DTYMK	0.000186	1.467488	1.200137	1.794397
ARSI	0.005963	1.269871	1.071036	1.50562
GPLD1	0.031803	0.743482	0.567215	0.974524
TYRP1	0.021102	0.833636	0.714194	0.973054
ATP13A4	0.001414	0.874713	0.805688	0.949651
KCNA3	0.014391	0.853699	0.752126	0.96899
CLCN6	0.018466	0.778543	0.632187	0.958781
ABCD1	0.003424	1.353191	1.10508	1.657007
TAP2	0.003728	1.292906	1.086858	1.538018
HIBADH	0.031123	1.281884	1.022782	1.606624
ACSL4	0.033403	1.182964	1.013297	1.381039
SLC11A2	0.044956	0.789247	0.626236	0.99469
KCNN4	0.0221	1.109325	1.015012	1.212403
MBOAT7	0.011825	1.350322	1.068761	1.706059
FUT1	0.002028	0.7315	0.599757	0.892181
GGT6	0.00108	0.873494	0.805452	0.947283
GUCY2C	0.022083	1.186862	1.024928	1.374382
GBE1	0.006437	1.328039	1.082877	1.628705
ACOXL	0.001951	0.776409	0.661526	0.911243
OAS3	0.004625	1.21391	1.061511	1.388189
KCNK3	0.006257	0.855864	0.765503	0.956891
SLC35D1	0.025534	1.297188	1.03235	1.629968
SLC5A6	0.02119	1.282518	1.037909	1.584774
ABCA6	0.015296	0.728558	0.564048	0.941049
SLC25A1	0.003222	1.416892	1.12368	1.786614
SLC3A2	0.000933	1.435106	1.158744	1.777381
SLC25A22	0.016864	1.33067	1.052712	1.68202

Genes	P.Value	HR	lower.95	upper.95
KCNK1	0.018367	1.154543	1.024561	1.301015
ADA	0.001057	1.294454	1.109185	1.51067
CACNA2D2	0.000438	0.877837	0.816337	0.943971
SORD	0.007735	1.286572	1.068818	1.54869
DERA	0.000199	1.517738	1.218255	1.890844
A4GALT	0.020678	1.166556	1.023839	1.329166
SDR42E1	0.04698	0.830436	0.691336	0.997523
CKMT2	0.027925	0.793279	0.645293	0.975202
CHST11	0.019896	1.208355	1.030394	1.417051
SUOX	0.042894	0.771664	0.600424	0.991742
PYGL	0.003232	1.236694	1.073629	1.424526
AMT	0.000356	0.775867	0.674982	0.89183
CHPF	0.008172	1.264472	1.062651	1.504624
GCLM	0.045436	1.146104	1.002773	1.309922
NUDT19	0.025577	1.271097	1.029725	1.569048
DHRS7	0.01717	1.276408	1.044281	1.560134
ATIC	0.000674	1.565253	1.208951	2.026564
NDUFS6	0.040549	1.230526	1.008968	1.500735
CYP24A1	0.023235	1.071303	1.009439	1.136959
PIK3CD	0.047135	0.845237	0.715947	0.997874
CPS1	0.000701	1.080616	1.033238	1.130166
HDC	0.004862	0.778278	0.653678	0.926629
HSD17B6	0.018628	0.897009	0.819363	0.982013
AHCY	0.032008	1.251041	1.019442	1.535256
SLC12A8	0.022261	1.224498	1.029298	1.456716
ABCA8	0.002406	0.778677	0.662513	0.91521
PGM5	0.007935	0.820368	0.7088	0.949496
ATP8B4	0.019851	0.724738	0.552744	0.95025
CDA	0.009304	1.097582	1.023204	1.177367
CH25H	0.008074	0.883388	0.805952	0.968264
GSTM5	0.016078	0.746262	0.588024	0.947081
SLC43A3	0.04568	0.871491	0.761505	0.997362
HPSE	0.021979	1.220726	1.029217	1.447869
SLC46A2	0.04921	0.898327	0.80729	0.999629
ENTPD2	0.005323	1.178193	1.049856	1.322218
UMPS	0.007527	1.451628	1.104478	1.907891
BEST4	0.013633	0.777015	0.635874	0.949484
ABCE1	0.015418	1.343401	1.057974	1.705831
HAL	0.026796	1.116375	1.012733	1.230624
GDPD1	0.027442	0.808778	0.669741	0.976677
NPC2	0.002992	0.836124	0.742935	0.941002
HGSNAT	0.023712	1.233983	1.028452	1.480589

Genes	P.Value	HR	lower.95	upper.95
RPE	0.000555	1.576146	1.217392	2.040621
ENPP4	0.016443	0.823792	0.703136	0.965152
PGD	0.00543	1.213463	1.058749	1.390785
SQLE	0.016465	1.183675	1.031307	1.358553
B4GALT4	0.000142	1.433487	1.190748	1.725709
EHHADH	0.044512	1.228814	1.005066	1.502373
CHIA	0.016925	0.896007	0.818797	0.980498
NDUFS2	0.004011	1.510288	1.140482	2.000005
SLC25A29	0.033746	0.852309	0.735405	0.987796
AGMAT	0.002069	1.291634	1.097536	1.520058
GMPS	0.000182	1.496323	1.211645	1.847886
OAS1	0.004586	1.197556	1.05721	1.356533
UGDH	0.03439	1.125225	1.00871	1.255198
DLD	0.022506	1.343895	1.04256	1.732325
DPEP2	0.004255	0.778517	0.655718	0.924312
KCNK17	0.018404	0.827485	0.706943	0.96858
PDXK	0.016206	1.324964	1.053372	1.66658
EXT1	0.000356	1.473696	1.191164	1.823242
MAOB	0.011501	0.863937	0.771292	0.967711
DHRSX	0.011665	1.28815	1.058059	1.568277
ITPK1	0.032085	1.301058	1.022772	1.655062
GANC	0.010028	0.738884	0.586864	0.930282
PDE10A	0.005161	1.209006	1.058434	1.380998
GOT1	0.033184	1.213381	1.015547	1.449755
FUCA2	0.000294	1.516189	1.210338	1.899329
ACBD5	0.043856	1.280229	1.006827	1.627873
SLC39A11	0.023186	1.28039	1.034368	1.584928
ACADSB	0.011487	0.811595	0.690297	0.954206
SLC27A4	0.001169	1.47721	1.167188	1.869577
SLC10A3	0.034493	1.305853	1.019666	1.672365
GGTLC1	0.000332	0.881942	0.82346	0.944577
LPCAT1	0.034368	0.88707	0.793866	0.991216
FOLR1	0.035871	0.933689	0.87573	0.995484
SCD	0.031347	1.14462	1.012163	1.294411
PSPH	0.005265	1.243506	1.067006	1.449202
CHIT1	0.017656	0.912579	0.84616	0.984211
CFTR	0.000732	0.838412	0.756894	0.92871
DPYSL2	0.001645	0.798302	0.69383	0.918506
AGPS	0.00893	1.392002	1.086347	1.783655
VDAC3	0.021395	1.269262	1.035966	1.555095
SCN1A	0.013044	0.751524	0.599792	0.941641
SLC15A1	0.005333	1.161124	1.045297	1.289787

**Genes**	**P.Value**	**HR**	**lower.95**	**upper.95**
B3GALNT1	0.011131	1.210072	1.044418	1.402
MMADHC	0.002135	1.509592	1.16065	1.963441
GGT2	0.027912	0.780257	0.625414	0.973435
ATP8B3	0.000244	1.272826	1.118926	1.447894
STARD9	0.025562	0.752456	0.586213	0.965843
POFUT1	0.02241	1.325165	1.040678	1.687421
CACNB1	0.002032	0.788754	0.678385	0.917079
OGDH	0.005298	1.432706	1.112736	1.844685
ETFB	0.027095	1.308698	1.030922	1.661317
CANT1	0.001923	1.391034	1.129187	1.713601
PDSS1	0.024793	1.303891	1.034223	1.643874
HSDL1	0.012452	0.736687	0.57969	0.936202
CYP27A1	0.014002	0.852658	0.750858	0.968259
PLA2G12B	0.017815	0.904571	0.832554	0.982817
AMDHD1	0.002099	1.275087	1.092166	1.488645
MAN1C1	0.049055	0.83732	0.701621	0.999265
GLRX2	0.000312	1.630073	1.249774	2.126094
SCN4B	0.002981	0.787997	0.673335	0.922185
GGH	0.004567	1.157014	1.046085	1.279707
HPRT1	0.010227	1.291073	1.062363	1.569022
NME4	0.002054	1.356848	1.117528	1.647419
NDUFB4	0.006227	1.409863	1.102289	1.80326
RRM1	0.002104	1.39018	1.126898	1.714975
KL	0.005391	0.765134	0.633648	0.923903
CYP4X1	0.014998	0.888387	0.807585	0.977275
AMPD1	0.001698	0.713206	0.577495	0.880809
HVCN1	0.034622	0.801436	0.652663	0.98412
GPD2	0.048556	1.254309	1.001438	1.571032
B3GNT5	0.000866	1.303157	1.115144	1.522869
KCNJ15	0.027355	0.90183	0.822739	0.988525
DHDH	0.017384	0.844388	0.73453	0.970677
PTGDS	0.007661	0.880408	0.801729	0.966808
ADSS	0.041324	1.326896	1.011194	1.741162
AQP5	0.034034	0.94611	0.898867	0.995835
SLC15A2	0.003732	0.843265	0.751494	0.946242
SCN7A	0.002137	0.824715	0.729254	0.932671
PNPLA3	0.023843	1.242982	1.029267	1.501072
GPD1L	0.000123	0.753001	0.651481	0.870342
GLB1L3	0.01644	0.902942	0.830682	0.981489
COQ10A	0.012361	0.716169	0.551348	0.930262
ACSL3	0.000532	1.392925	1.154777	1.680185
ALDH16A1	0.018492	1.337855	1.050103	1.704457

Genes	P.Value	HR	lower.95	upper.95
GSTA1	0.034927	0.936627	0.881343	0.995378
SLC6A14	0.019357	1.096959	1.015095	1.185424
ACSS1	0.000903	0.796887	0.696903	0.911216
GLB1L2	0.0294	0.8797	0.783869	0.987246
ITPKC	0.002947	1.347926	1.107103	1.641133
ADHFE1	0.004031	0.795825	0.681111	0.929858
UQCRQ	0.020374	1.280272	1.039031	1.577525
EPHX1	0.001801	0.834995	0.7456	0.935107
COX8A	0.003597	1.471478	1.13455	1.908464
LPGAT1	0.000601	1.433619	1.167024	1.761115
CSGALNACT1	0.011474	1.241501	1.049802	1.468205
LBR	0.009744	1.330166	1.0714	1.651431
PNPLA7	0.002788	0.772064	0.651649	0.914728
CEL	0.038478	0.851667	0.731539	0.991521
CACNA1D	0.018098	0.835191	0.719331	0.969712
SMPD4	0.01145	1.388575	1.076622	1.790917
PAOX	0.012915	0.719672	0.55527	0.93275
PRDX6	0.023743	1.325111	1.038219	1.691279
SLC16A11	0.003236	0.801675	0.69198	0.92876
GALNT14	0.028902	1.104297	1.010263	1.207084
SLC39A1	0.015043	1.365341	1.062234	1.754937
B3GNT8	0.005026	0.864014	0.780136	0.956909
B3GALT2	0.001183	0.763626	0.648782	0.898799
GART	0.017544	1.331316	1.051292	1.685927
ATP2B1	0.006869	1.240055	1.060938	1.449412
PLCB2	0.041934	0.857497	0.739436	0.994407
LDHB	0.031238	1.161965	1.013616	1.332027
HSD17B13	0.01148	0.841596	0.736258	0.962004
FA2H	0.00969	1.14258	1.032822	1.264002
GNMT	0.000645	0.702042	0.572934	0.860243
CNGA3	0.017545	0.875821	0.785047	0.977092
SLC7A11	0.012917	1.112852	1.022885	1.210732
ELOVL2	0.014796	1.212373	1.038439	1.41544
ITPKB	0.046582	0.829126	0.689402	0.997169
TAP1	0.017337	1.176723	1.029107	1.345514
SLC7A5	0.003146	1.184663	1.058646	1.32568
ALG10	0.039289	1.270683	1.01183	1.595759
ABCC6	0.009685	0.832543	0.724607	0.956557
NUDT15	0.001785	1.478201	1.156748	1.888982
COX5A	0.003651	1.439409	1.12597	1.840101
GOT2	0.029351	1.337037	1.029596	1.736282
ACAT2	0.001053	1.379889	1.138093	1.673058

Genes	P.Value	HR	lower.95	upper.95
TTYH3	0.031659	1.19842	1.016025	1.413557
ABCA12	0.030643	1.127817	1.011288	1.257774
ABHD4	0.003892	1.346322	1.10018	1.647534
HK2	0.004139	1.196138	1.058314	1.351911
SLC39A14	0.027429	1.196223	1.020137	1.402703
COQ3	0.035742	1.284029	1.016794	1.621499
ENPP1	0.002341	1.245871	1.081391	1.435368
ITPKA	0.011284	1.100022	1.021824	1.184204
HSD3B7	0.038814	1.216016	1.010102	1.463906
PGM1	0.011225	1.334532	1.067709	1.668034
ABCA3	0.000975	0.872454	0.804495	0.946153
SLC35F2	0.01256	1.238251	1.046964	1.464487
BDH2	0.012919	0.762635	0.615935	0.944276
MGST3	0.007618	1.443162	1.102307	1.889417
PGAM1	0.000303	1.520577	1.211345	1.908751
MTHFD1L	0.005957	1.357965	1.091898	1.688865
GALNT3	0.008289	1.186671	1.045077	1.347449
COX6B1	0.007553	1.313868	1.075407	1.605205
GALNT13	0.041674	1.150302	1.005294	1.316227
MICAL3	0.025341	1.258652	1.028822	1.539825
MTHFD1	0.000143	1.573993	1.245809	1.98863
SLC38A2	0.025076	1.254999	1.028823	1.530897
SDHA	0.028112	1.294616	1.028113	1.6302
CAT	0.01485	0.808987	0.682139	0.959422
APRT	0.028415	1.271172	1.025675	1.575429
ENTPD1	0.046166	0.793822	0.632633	0.996081
CHST14	0.03727	1.321393	1.016571	1.717617
GPX3	0.026151	0.867213	0.764885	0.98323
SLC35E4	0.02253	1.360527	1.044322	1.772473
PLCG2	0.025069	0.806105	0.667572	0.973386
SLC1A5	0.044307	1.230802	1.005302	1.506885
TXNRD1	0.002885	1.151035	1.049316	1.262614
SLC25A16	0.01817	0.758883	0.603602	0.954112
ADCY9	0.013614	0.822371	0.704043	0.960586
KCTD3	0.006315	1.269827	1.069765	1.507304
CERKL	0.005045	0.790519	0.670747	0.931679
FDPS	0.002304	1.430185	1.136238	1.800177
SLC7A8	0.026686	0.874527	0.77674	0.984625
ELOVL6	0.001236	1.208357	1.077275	1.355388
SLCO4C1	0.01811	0.88202	0.794809	0.9788
