# Supplementary material for: Systematically analyzed molecular characteristics of lung adenocarcinoma using metabolism-related genes classification
Source: Genet Mol Biol. 2023 Jan 6;45(4):e20220121. doi: 10.1590/1678-4685-GMB-2022-0121 (PMC9830935; doi:10.1590/1678-4685-GMB-2022-0121)
Supplement: Table S3 - [file 1415-4757-GMB-45-4-e20220121-s14.pdf]

Genetics and Molecular Biology
https://doi.org/ 10.1590/1678-4685-GMB-2022-0121

Supplementary Material to “Systematically analyzed molecular characteristics of lung adenocarcinoma using metabolism-related genes classification”

Table S3. The association between molecular clusters and clinical signatures by multivariable Cox regression analysis.

Factors	HR	lower.95	upper.95	p.value
molecular clusters	1.231	0.988	1.023	0.031
Gender	0.912	0.645	1.289	0.602
T.Stage	1.253	1	1.571	0.050
N.Stage	1.256	0.89	1.774	0.049
M.Stage	0.871	0.319	2.377	0.787
Stage	1.367	0.913	2.047	0.012
