# Supplementary material for: Systematically analyzed molecular characteristics of lung adenocarcinoma using metabolism-related genes classification
Source: Genet Mol Biol. 2023 Jan 6;45(4):e20220121. doi: 10.1590/1678-4685-GMB-2022-0121 (PMC9830935; doi:10.1590/1678-4685-GMB-2022-0121)
Supplement: Table S4 - [file 1415-4757-GMB-45-4-e20220121-s15.pdf]

Genetics and Molecular Biology
https://doi.org/ 10.1590/1678-4685-GMB-2022-0121

Supplementary Material to "Systematically analyzed molecular characteristics of lung adenocarcinoma using metabolism-related genes classification"

Table S4. The association between 60 genes classifier and clinical signatures by univariate Cox regression analysis.

Factors	HR	lower.95	upper.95	p.value
Age	1.008	0.993	1.024	0.294
Gender	1.049	0.784	1.405	0.747
T.Stage	1.523	1.265	1.834	0.000
N.Stage	1.695	1.429	2.011	0.000
M.Stage	2.133	1.245	3.654	0.006
Stage	1.673	1.456	1.921	0.000
Classifier	1.714	1.402	2.095	0.000
